# Conformational diversity in purified prions produced *in vitro*

**DOI:** 10.1371/journal.ppat.1011083

**Published:** 2023-01-10

**Authors:** Daniel J. Walsh, Abigail M. Schwind, Geoffrey P. Noble, Surachai Supattapone

**Affiliations:** 1 Department of Biochemistry Geisel School of Medicine at Dartmouth, Hanover, New Hampshire, United States of America; 2 Department of Medicine, Geisel School of Medicine at Dartmouth, Hanover, New Hampshire, United States of America; University of Massachusetts Amherst, UNITED STATES

## Abstract

Prion diseases are caused by misfolding of either wild-type or mutant forms of the prion protein (PrP) into self-propagating, pathogenic conformers, collectively termed PrP^Sc^. Both wild-type and mutant PrP^Sc^ molecules exhibit conformational diversity *in vivo*, but purified prions generated by the serial protein misfolding cyclic amplification (sPMCA) technique do not display this same diversity *in vitro*. This discrepancy has left a gap in our understanding of how conformational diversity arises at the molecular level in both types of prions. Here, we use continuous shaking instead of sPMCA to generate conformationally diverse purified prions *in vitro*. Using this approach, we show for the first time that wild type prions initially seeded by different native strains can propagate as metastable PrP^Sc^ conformers with distinguishable strain properties in purified reactions containing a single active cofactor. Propagation of these metastable PrP^Sc^ conformers requires appropriate shaking conditions, and changes in these conditions cause all the different PrP^Sc^ conformers to converge irreversibly into the same single conformer as that produced in sPMCA reactions. We also use continuous shaking to show that two mutant PrP molecules with different pathogenic point mutations (D177N and E199K) adopt distinguishable PrP^Sc^ conformations in reactions containing pure protein substrate without cofactors. Unlike wild-type prions, the conformations of mutant prions appear to be dictated by substrate sequence rather than seed conformation. Overall, our studies using purified substrates in shaking reactions show that wild-type and mutant prions use fundamentally different mechanisms to generate conformational diversity at the molecular level.

## Author summary

Prions are misfolded proteins that cause infectious, inherited, and sporadic diseases in many animals, including humans. Prions can misfold into a variety of distinct misfolded shapes, but we do not yet know the molecular mechanisms that generate this diversity. We report that shaking test tube reactions with purified components can be used to study the mechanisms by which prions with diverse shapes are formed. Our results show that infectious (wild-type) prions and inherited (mutant) prions use different mechanisms to produce diverse misfolded shapes.

## Introduction

Mammalian prion diseases are fatal neurodegenerative diseases that can occur in inherited, sporadic, or infectious forms. All three forms of prion disease are caused by misfolding of a specific membrane-bound glycoprotein termed the prion protein (PrP), which is most highly expressed in neurons [1]. Examples of inherited prion diseases in humans include fatal familial insomnia (FFI), familial Creutzfeldt-Jakob disease (fCJD), and Gerstmann-Sträussler-Scheinker syndrome (GSS), all of which have been linked to specific PrP mutations that promote misfolding [2]. Notably, patients with FFI, fCJD, and GSS display distinct misfolded mutant PrP conformations, neuropathological profiles, and clinical symptoms. In contrast to the inherited forms of prion disease, sporadic and infectious forms such as sporadic Creutzfeldt-Jakob disease (sCJD) and kuru are not associated with PrP mutations. Instead, they are caused by the misfolding of wild-type PrP^C^ (the normal conformer of PrP) into self-replicating amyloid conformers collectively termed PrP^Sc^ [1,3–5]. Furthermore, different self-replicating wild-type PrP^Sc^ conformers are associated with specific infectious strain properties, including distinctive neuropathological and clinical profiles [6].

Thus, both wild-type and mutant PrP molecules can form a variety of specific misfolded conformers, each of which causes its own pattern of disease. However, our understanding of the molecular mechanisms responsible for the misfolding and self-propagation of wild-type and mutant PrP into specific pathogenic conformers remains incomplete. Much of our current knowledge about the molecular mechanism of prion replication has been gained by using the serial Protein Misfolding Cyclic Amplification (sPMCA) technique, in which infectious prions can be generated and serially propagated *in vitro* through a process that uses intermittent sonication to fragment PrP^Sc^ fibrils [7].

Using reconstituted sPMCA reactions, we and others have shown that cofactor molecules such as phospholipids and polyanions are required to generate wild-type infectious prions *in vitro*[8–13]. Moreover, specific cofactor molecules appear to dictate strain properties in purified sPMCA reactions, regardless of input seed [10,12–14]. Taken together, these results strongly suggest that each prion strain requires its own specific endogenous cofactor and neurotropism could be explained by variation in the levels of strain-specific cofactors in different brain regions [15–17]. However, it has been difficult to reconcile this “cofactor selection” model with (1) the phenomena of strain adaptation, in which strain properties shift gradually upon inoculation into a new host species rather than immediately [18–20] and (2) the presence of multiple conformational variants or “quasi-species” in the brains of animals infected with a single prion strain [19,21–23].

sPMCA experiments have also been used to show that purified mutant E199K and D177N mouse (Mo) recombinant (rec)PrP molecules can spontaneously misfold into PrP^Sc^ molecules in the absence of cofactors, but cofactor molecules are required for these mutant PrP^Sc^ molecules to subsequently seed wild-type recPrP substrate[24]. However, the pure E199K and D177N mutant PrP^Sc^ molecules produced in these sPMCA experiments show very similar patterns in western blots following protease digestion [24]. In contrast, the western blot patterns of homologous human (Hu) E200K and D178N PrP^Sc^ molecules are easily distinguishable in patients with fCJD and FFI, respectively [25]. This discrepancy raises the question as to whether different mutant PrP molecules can independently adopt their specific disease-associated PrP^Sc^ conformations, or whether additional cellular factors might be required to facilitate the formation of specific PrP^Sc^ conformations.

One potential problem with using sPMCA to study mechanistic questions is that it may amplify prions too efficiently to capture metastable conformations. Intermittent sonication drastically accelerates the prion replication process, which may cause metastable conformational variants to quickly evolve into a single (most rapidly replicating) PrP^Sc^ conformation, making it difficult to detect mechanistically relevant states such as partially-adapted conformers, quasi-species, and distinguishable mutant PrP^Sc^ states. To overcome this potential problem, we decided to investigate how continuous shaking, which is gentler and less efficient than PMCA, impacts the conformational landscape of wild-type and mutant prions produced in chemically-defined reactions.

## Results

### Propagation of different wild-type mouse PrP^Sc^ conformers in purified shaking reactions

**Fig 1:**
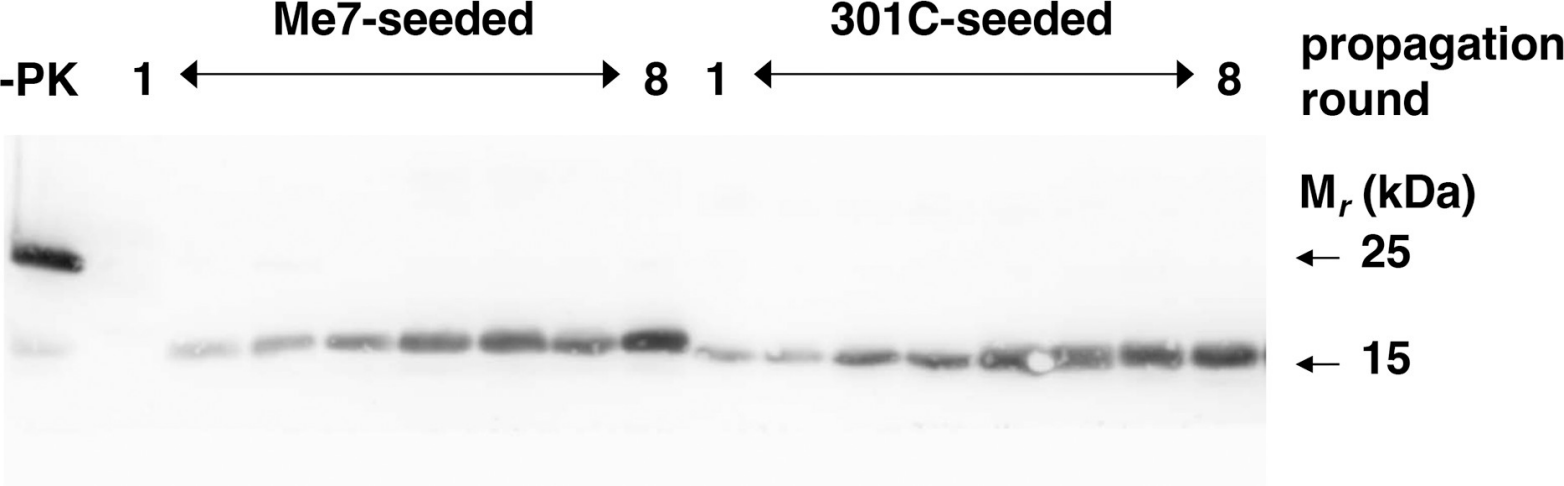
Shaking-generated wild-type Mo recPrP^Sc^ molecules. Western blot showing rounds 1–8 of serial propagation shaking reactions containing wild-type Mo recPrP substrate plus purified brain phospholipid cofactor initially seeded with Me7 or 301C brain homogenate, as indicated. (-PK) = sample not subjected to proteinase K digestion; all other samples were proteolyzed.

We previously reported that purified sPMCA reactions containing only Mo recPrP substrate plus either synthetic phosphatidylethanolamine (PE) cofactor or purified brain phospholipid cofactor preparation containing PE as the only active cofactor produced a self-replicating synthetic prion strain termed “cofactor PrP^Sc^,” which has a proteinase K (PK)-resistant core of ~18 kDa and a level of specific infectivity ~(2.2 x 10^5^ LD_50_/μg PrP^Sc^) similar to that of brain-derived PrP^Sc^ [10,26]. Strikingly, the same cofactor PrP^Sc^ strain is invariably produced in purified sPMCA reactions, even when these reactions are initially seeded by different prion strains [10]. Specifically, the products of such reactions displayed similar biochemical and infectious properties, including identical neuropathological profiles in bioassays [10]. Here, we seeded a purified substrate cocktail (Mo recPrP and purified brain phospholipid cofactor) with either of two different mouse scrapie strains (Me7 and 301C), subjected the reactions to continuous shaking in a commercial Ohaus shaker for 3 days at 37°C instead of sPMCA, and performed subsequent cycles of serial propagation at a 1:5 dilution. Unlike our results with sPMCA [10], shaking reactions initially seeded with these two different prion strains produced and serially propagated PrP^Sc^ conformers with easily distinguishable PK-resistant bands **(Fig 1)**. Specifically, the PK-resistant core of PrP^Sc^ molecules in shaking reactions initially seeded with 301C is smaller than that of PrP^Sc^ molecules produced in reactions initially seeded with Me7 **(Figs 1 and S1)**, similar to the pattern observed for brain-derived PrP^Sc^ molecules in mice infected with these two strains [10].

### Bioassay and neuropathology of mice inoculated with wild-type mouse PrP^Sc^ conformers produced in purified shaking reactions

**Fig 2:**
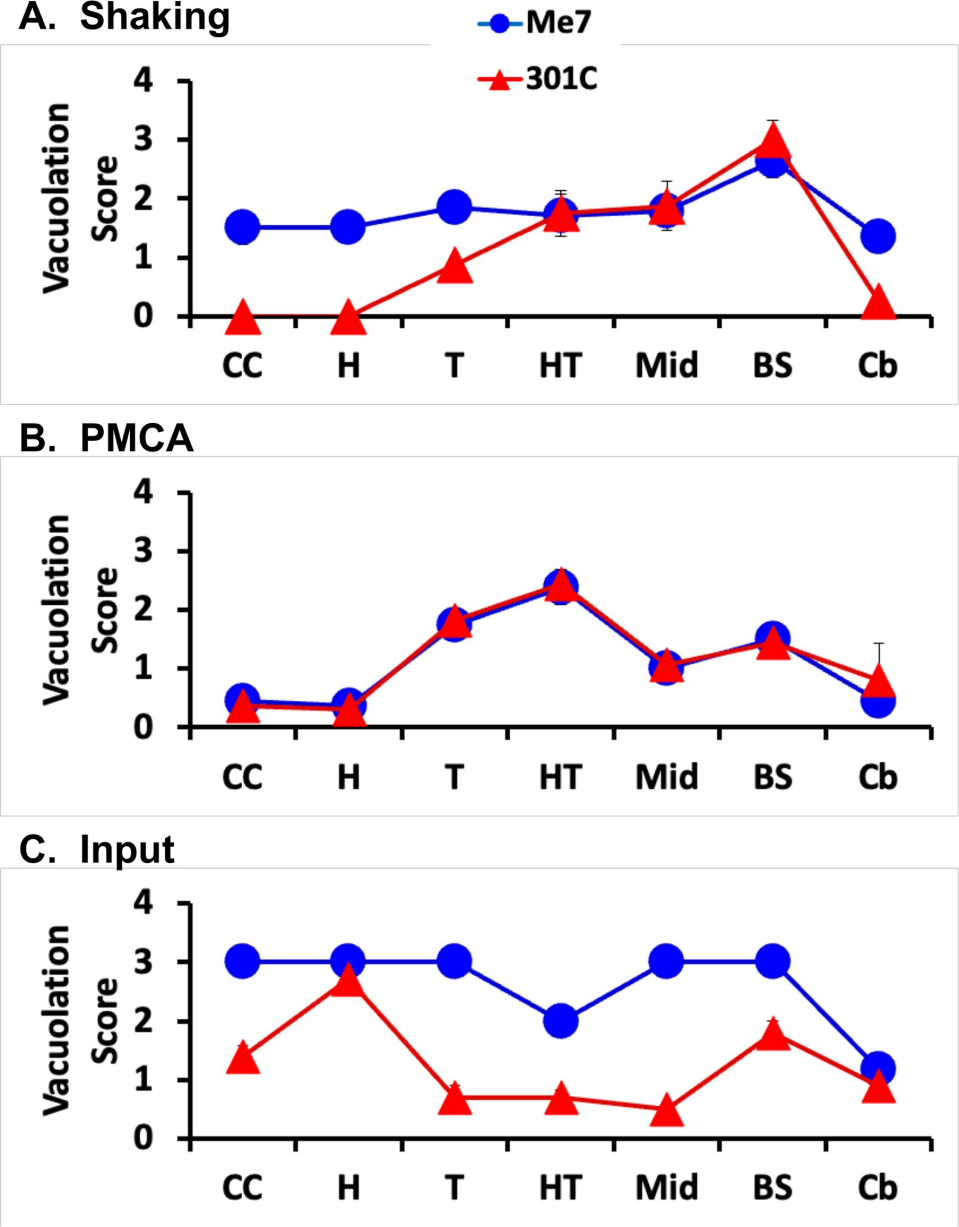
Regional neuropathology of infected mice. Profiles of vacuolation scores of animals inoculated with samples containing **A.** Shaking-propagated Mo recPrP^Sc^ prions, **B.** PMCA-propagated Mo recPrP^Sc^ molecules, or **C.** input prions. Prion strains: Me7 = blue circles, 301C = red triangles. Brain regions: CC = cerebral cortex (all layers), H = hippocampus, T = Thalamus, HT = Hypothalamus, Mid = Midbrain, BS = brain stem, Cb = cerebellum. The mean values (n = 4–8) are shown ± S.E.M.

To examine the infectious strain properties of these two different shaking PrP^Sc^ products produced with the same cofactor, we performed 26 serial propagation cycles to ensure that all of the PrP^Sc^ molecules in the initial seeds were fully removed by dilution and intracerebrally injected the final round 26 products into wild-type C57BL mice. Both of the purified shaking products caused scrapie with a similar incubation time of ~410 days **(S2 Fig)**, and no differences in the glycosylation pattern or migration of PK-resistant PrP^Sc^ molecules in the brains of mice infected with the two different shaking products could be observed **(S3 Fig)**. However, we did observe numerous differences in regional neuropathology between mice infected with the Me7-seeded shaking product **(Fig 2A, blue circles)** versus the 301C-seeded shaking product **(Fig 2A, red triangles)**. Differences in the degree of vacuolation induced by the two different shaking products were most easily observed in the cerebral cortex, hippocampus, and cerebellum **(Fig 2A and S4 Fig, top 3 rows)**. In contrast, sPMCA products of identical reaction mixtures seeded with either Me7 or 301C produced identical neuropathological profiles [10] **(Fig 2B, compare blue circles and red triangles)**. Notably, the neuropathological profiles for both of the shaking PrP^Sc^ products **(Fig 2A)** were different from the profiles induced either by sPMCA products **(Fig 2B)** or input seeds [10] **(Fig 2C)**. These results reveal for the first time that seed-dependent conformational differences can be generated and propagated in reactions containing recPrP and purified cofactor.

### Propagation of different wild-type bank vole PrP^Sc^ conformers in purified shaking reactions

**Fig 3:**
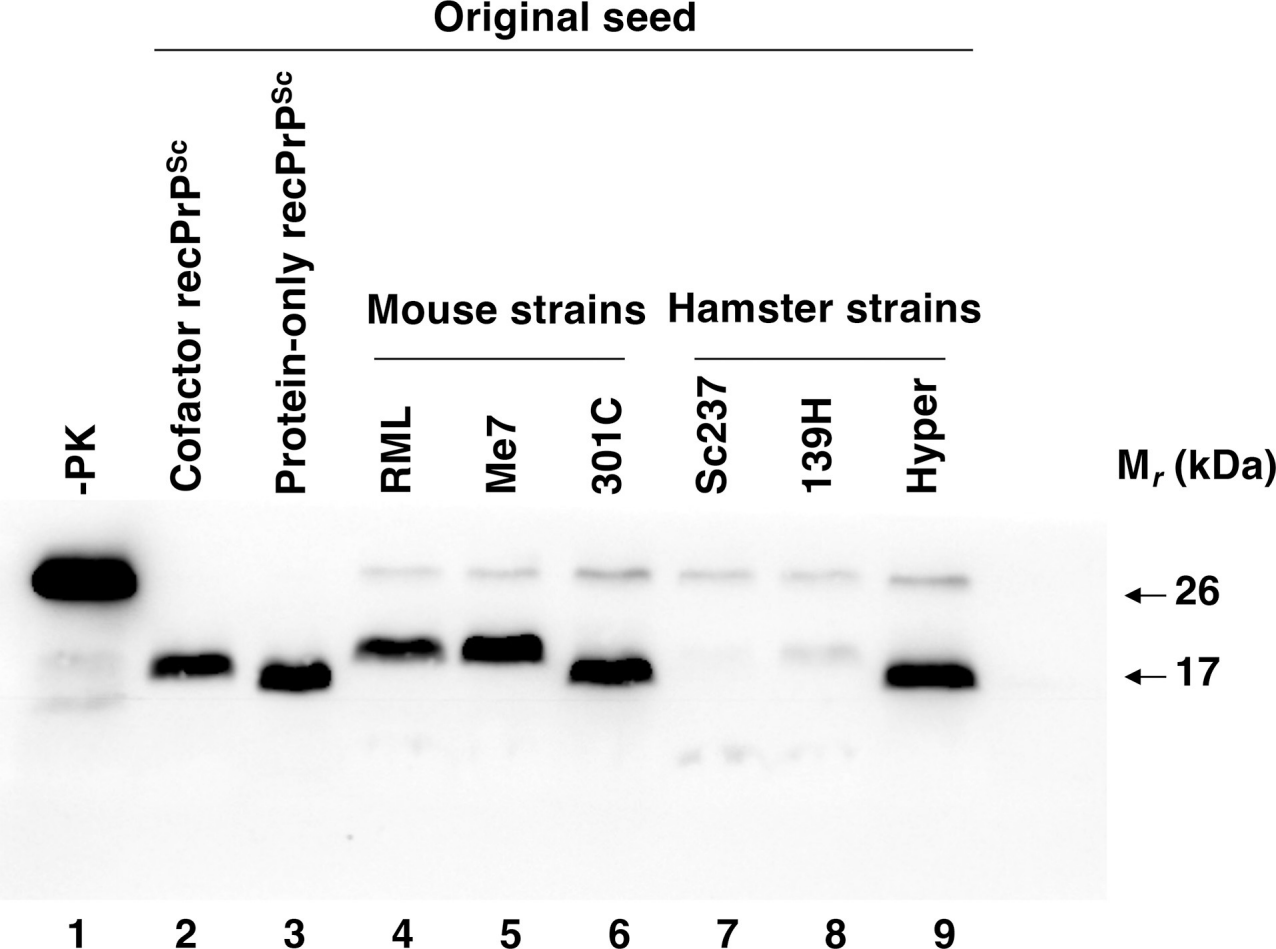
Shaking-generated wild-type BV recPrP^Sc^ molecules. Western blot showing the final product of 26-round serial propagation shaking reactions containing wild-type BV recPrP substrate plus purified brain phospholipid cofactor. Each sample was initially seeded with sPMCA-generated recPrP^Sc^ seeds (lanes 2–3), native mouse prions (4–6), or native hamster prions (lanes 7–9), as indicated. (-PK) = sample not subjected to proteinase K digestion; all other samples were proteolyzed.

Bank vole (BV) PrP can be seeded by a wider variety of prion strains from different animal species than Mo PrP [16]. We therefore repeated our experiment using BV rather than Mo PrP substrate to investigate whether conformational differences could also be generated and maintained in purified substrate cocktails initially seeded with hamster as well as mouse prion strains. Six lines of substrate cocktails containing wild-type BV recPrP plus purified phospholipid cofactor were initially seeded with either mouse (RML, Me7, or 301C) or hamster (Sc237, 139H, or Hyper) prions, subjected to shaking at 2,000 rpm, and serially propagated for 26 cycles as before. A western blot of the final (round 26) shaking products shows that BV recPrP^Sc^ molecules initially seeded with 301C have a smaller PK-resistant core than BV recPrP^Sc^ molecules initially seeded with Me7 **(Fig 3, compare lanes 5 and 6)**, similar to the results obtained for Mo recPrP^Sc^ molecules initially seeded with these same two strains **(Fig 1)**. Overall, a diverse set of PK-resistant banding patterns were observed among the shaking PrP^Sc^ products initially seeded with natural mouse and hamster strains **(Fig 3, lanes 4–9, and S1 Fig)**. For example, two of the shaking PrP^Sc^ products initially seeded with hamster strains (Sc237 and 139H) displayed much less PK-resistance than the other shaking PrP^Sc^ products **(Fig 3, lanes 7–8)**, and some of the shaking PrP^Sc^ products (e.g. RML, Me7, 139H) had PK-resistant cores that were ~2 kDa larger than the PK-resistant core of the shaking PrP^Sc^ product initially seeded with sPMCA-generated cofactor recPrP^Sc^ **(Fig 3, compare lanes 4,5, and 8 to lane 2)**. These results showing the ability of a purified cofactor to facilitate propagation of wide variety of BV PrP^Sc^ conformers in shaking reactions confirm and extend our original observation with Me7- and 301C-seeded Mo PrP^Sc^ conformers.

### Altered shaking conditions facilitate convergence of different cofactor PrP^Sc^ conformers

**Fig 4:**
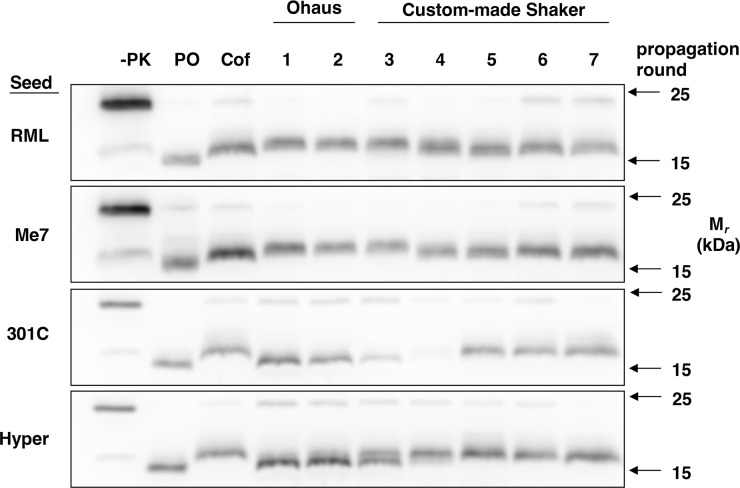
Effect of different shaking conditions on BV recPrP^Sc^ variants. Western blots showing adaptation of BV recPrP^Sc^ variants during serial propagation at lower shaking speed. All reactions were propagated in a substrate cocktail containing BV recPrP plus purified phospholipid cofactor. Reactions were initially seeded with RML, Me7, 301C, or Hyper as indicated. Blots show two rounds of serial propagation with continuous shaking in a commercial Ohaus shaker with 3 mm orbit at 2,000 rpm followed by 5 additional rounds of serial propagation in a custom-made shaker with 8 mm orbit at 1,200 rpm. Reference shaking-generated protein-only PrP^Sc^ (PO) and cofactor PrP^Sc^ (Cof) samples are included on each blot for MW comparison. (-PK) = Custom-made shaker round 7 samples not subject to proteinase K digestion; all other samples were proteolyzed.

Having established high-speed shaking conditions that are able to maintain seed-dependent PrP^Sc^ conformational differences in substrate cocktails containing wild-type recPrP and purified cofactor, we next examined the effect of varying shaking conditions on our ability to maintain different PrP^Sc^ conformations in purified reactions. In one of these experiments, we changed from a commercial Ohaus shaker with 3 mm orbit at 2,000 rpm to a custom-made shaker with 8 mm orbit at ~1,200 rpm during serial propagation **(Fig 4)**. Interestingly, all four of the shaking PrP^Sc^ products initially seeded with native strains gradually converged into a single conformation with a PK-resistant core similar to that of sPMCA-generated cofactor PrP^Sc^ within 4 rounds of serial propagation **(Fig 4, compare last lane with 3**^**rd**^ **lane (Cof) on all 4 blots)**. It is notable that the two shaking PrP^Sc^ products with PK-resistant cores that were initially larger than cofactor PrP^Sc^ (RML and Me7) gradually shifted downwards at lower shaking speed **(Fig 4, top two blots)** whereas the two shaking PrP^Sc^ products with PK-resistant cores that were initially smaller than cofactor PrP^Sc^ (301C and Hyper) gradually shifted upwards at lower shaking speed **(Fig 4, bottom two blots)**. These shifts could not be stably reversed by subsequent propagation in the Ohaus shaker **(S5 Fig)**. These results suggest that changing the physical parameters of continuous shaking of purified PrP^Sc^ propagation reactions can lead to a similar result as sPMCA, i.e. irreversible convergence of multiple strains into a single conformation (cofactor PrP^Sc^) [10]. These results indicate that most of the PrP^Sc^ conformers formed by continuous shaking are not thermodynamically as stable as cofactor PrP^Sc^ and can only be maintained under specific shaking conditions.

### The primary sequence of mutant PrP substrate molecules determines PrP^Sc^ conformation in shaking reactions

**Fig 5:**
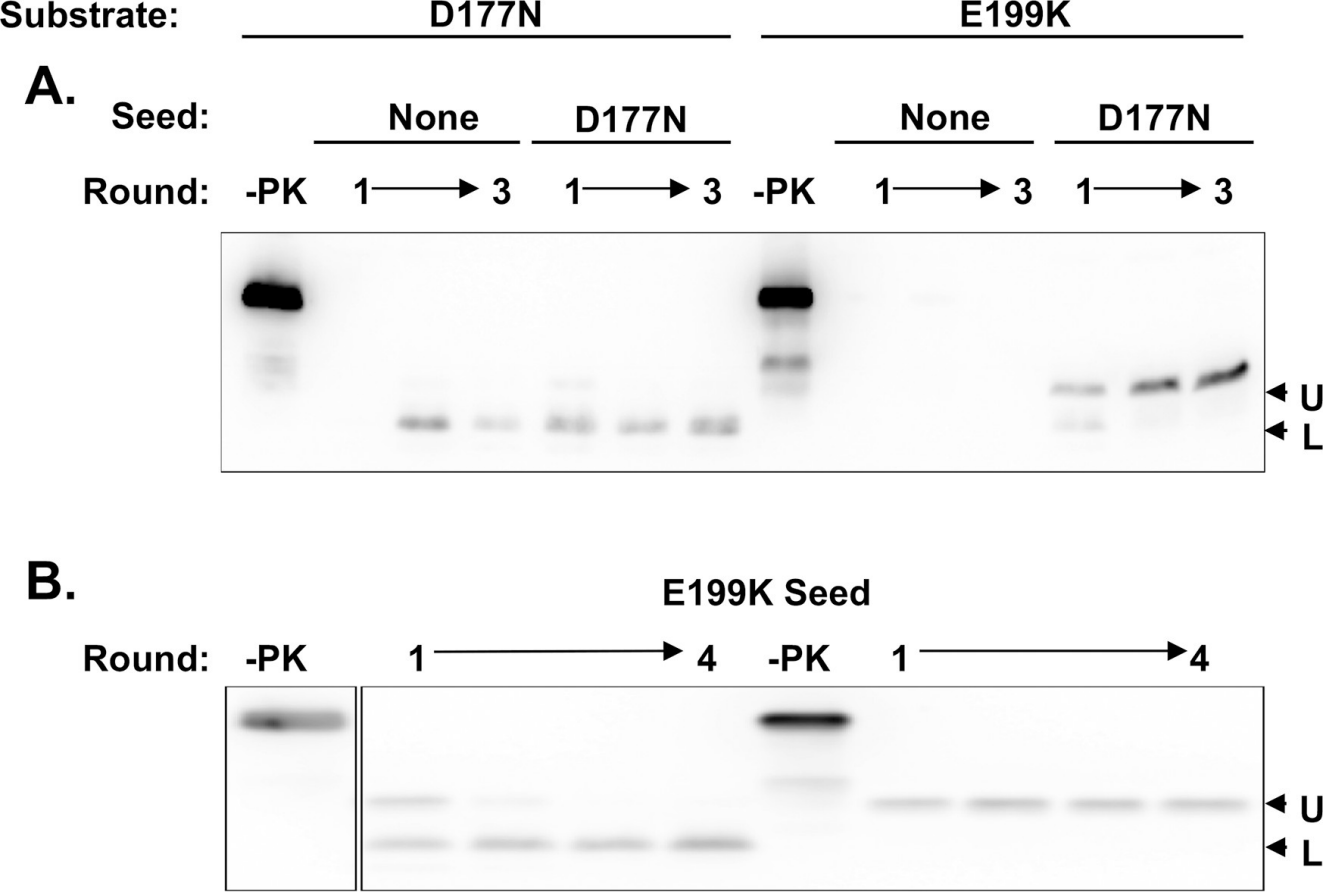
Shaking-induced mutant recPrP^Sc^ molecules. Western blots showing serially propagated shaking conversion reactions using D177N (left) and E199K (right) mutant Mo recPrP substrates. Reactions were initially seeded with **(A)** buffer (none), PMCA-generated D177N Mo PrP^Sc^, or **(B)** shaking-generated E199K Mo PrP^Sc^, as indicated. (-PK) = sample not subjected to proteinase K digestion; all other samples were proteolyzed. U = Upper MW band (~16 kDa); L = Lower MW band (~10 kDa).

We previously showed that both D177N and E199K Mo recPrP molecules can spontaneously misfold into self-propagating mutant PrP^Sc^ molecules in sPMCA reactions containing pure protein substrate without cofactors [24]. However, analysis by PK digestion could not distinguish between the two sPMCA products as both mutant PrP^Sc^ molecules produced similar doublet patterns consisting of two PK-resistant bands with MW ~16 kDa and ~10 KDa [24]. Here, we subjected these same pure mutant recPrP substrates to continuous shaking instead of sPMCA **(Fig 5)**. In shaking reactions, D177N substrate produces a mutant PrP^Sc^ conformer that predominantly shows the ~10 kDa lower MW PK-resistant band (L), whereas E199K substrate produces a mutant PrP^Sc^ conformer that predominantly shows the ~16 kDa upper MW PK-resistant band (U) **(Fig 5)**. Remarkably, each substrate appears to produce its specific PrP^Sc^ conformer regardless of whether the reactions are unseeded **(Fig 5A)**, seeded with D177N PrP^Sc^ **(Fig 5A)**, or seeded with E199K PrP^Sc^ **(Fig 5B)**. The only exception to this general observation is that unseeded E199K substrate does not appear to spontaneously produce mutant PrP^Sc^ in shaking reactions. The specificity revealed in these continuous shaking experiments (which was not previously evident in sPMCA experiments) show for the first time that the primary sequence of mutant PrP substrates, rather than seed conformation, dictates the conformation of product PrP^Sc^ molecules in purified shaking reactions.

## Discussion

In this manuscript, we have used continuous shaking instead of sPMCA to study both wild-type and mutant prion formation in purified conversion reactions. This alternative approach provided two important new insights about the mechanism of prion formation that could not be obtained in sPMCA experiments: (1) a wide variety of different metastable wild-type PrP^Sc^ conformers can propagate in the presence of a single active cofactor, and (2) the specific conformations of mutant PrP^Sc^ molecules can be dictated by primary sequence rather than seed conformation.

### Different metastable wild-type PrP^Sc^ conformers can propagate in the presence of a single active cofactor

Several studies in prion-infected cells and animals suggest that individual prion strains contain a “cloud” of different PrP^Sc^ conformers termed quasi-species, and that strain adaptation occurs by evolutionary selection of a favored conformation in the new host [21–23]. From this, we can reasonably infer that the fidelity of PrP^Sc^ replication must be slightly imperfect to allow generation of quasi-species in animals infected with a single prion strain.

However, previous work showed that only one PrP^Sc^ conformation and infectious strain can be formed when only a single purified cofactor is provided in sPMCA reactions [10,27], and different strains are produced in sPMCA reactions using different pure cofactors [12]. Furthermore, different prion strains appear to have different cofactor and glycosylation preferences for serial propagation in sPMCA reactions [16,28]. Collectively, these observations support a “cofactor selection” model of strain diversity in which each prion strain requires a unique cofactor to faithfully propagate its specific PrP^Sc^ structure [15,16,29].

Here, we report for the first time that several different PrP^Sc^ conformations can be faithfully propagated in continuous shaking reactions containing purified phospholipid cofactor. It appears that these different PrP^Sc^ conformers can be maintained indefinitely under specific shaking conditions; we have been able to propagate all the conformers reported here for a minimum 26 rounds, after which all of the PrP^Sc^ molecules from the original seed should be completely removed by serial dilution. Although we used different native prion strains as initial seeds to generate these new PrP^Sc^ conformers, the resulting shaking-produced prions do not appear to have the same strain properties (neuropathological profile) as the original native seed strain. Moreover, all of these distinct PrP^Sc^ conformers stochastically adapt into the single active cofactor PrP^Sc^ conformation under different shaking conditions. Therefore, we conclude that the different PrP^Sc^ conformers produced with purified cofactor substrate by shaking are metastable, i.e. they can be faithfully propagated under specific shaking conditions but are thermodynamically predisposed to converge into a single conformation under different shaking conditions or in sPMCA reactions. Presumably, these alternative conditions allow the most thermodynamically favored conformation (i.e. cofactor PrP^Sc^) to out-compete alternate conformations during serial propagation.

Most importantly, the detection of metastable PrP^Sc^ conformers with a purified cofactor is consistent with the proposal that PrP^Sc^ replication process may give rise to quasi-species that form a cloud of conformers in a single prion strain. Our results suggest that a cloud of different PrP^Sc^ conformers could be formed even when the replication process uses a single active cofactor. As previously proposed by Collinge and colleagues [22], such conformational clouds allow a pure prion strain to undergo adaptation by evolution. At the same time, because these PrP^Sc^ conformers are metastable (predisposed to converge into a single conformation that is dictated by the cofactor), the cofactor selection model still provides the best explanation for characteristic strain-specific properties such as selective neurotropism [15,16,29]. Thus, our discovery that different metastable prion strains can be formed with purified cofactor reconciles the existence of PrP^Sc^ quasi-species with the cofactor selection model strain diversity.

### Specific conformations of mutant PrP^Sc^ molecules can be dictated by primary sequence rather than seed conformation

In previous studies, we found that the protease digestion patterns of D177N and E199K Mo PrP^Sc^ molecules generated by sPMCA could not be distinguished from each other [24]. Here, we observed for the first time that pure D177N and E199K Mo PrP molecules adopt distinct mutant PrP^Sc^ conformations with different protease-resistant cores that are easily distinguished by western blot and mass-spectrometry. Notably, the PK-resistant core of shaking-induced D177N Mo PrP^Sc^ is smaller than that of E199K Mo PrP^Sc^, similar to the pattern seen in patient brains and transgenic mice containing D178N and E200K HuPrP^Sc^ molecules [25].

There are two novel and mechanistically significant aspects of this observation. First, the different mutant PrP^Sc^ conformers are formed with pure protein substrates in the absence of cofactors. Second, the mutant PrP^Sc^ conformations appear to be dictated solely by the primary sequence of the mutant PrP substrate rather than the seed. This point is most clearly shown in cross-seeding experiments in which each mutant PrP substrate misfolds into its own sequence-specific PrP^Sc^ conformation instead of faithfully propagating the seed PrP^Sc^ conformation. In addition, pure D177N substrate also folds into its characteristic PrP^Sc^ conformation spontaneously in unseeded reactions. Overall, our results show that the specific conformations of mutant PrP^Sc^ molecules are intrinsic properties of mutant PrP substrates.

More generally, these observations reveal the two key differences in the way that wild-type and mutant PrP^Sc^ molecules are formed. First, whereas the conformation of wild-type prions is dictated by cofactor molecules and the conformation of the input seed, the conformation of mutant prions appears to be determined only by the primary sequence of the mutant PrP substrate. Second, whereas the *in vitro* formation of different wild-type prions is cofactor-dependent, the formation of mutant prions with different conformations does not require any accessory (non-PrP) molecules.

### Using continuous shaking to study prion formation mechanisms

We and others have previously used continuous shaking as an alternative to PMCA to generate prions *in vitro* [30–32]. In our experience, the process of PrP^Sc^ replication appears to be significantly slower and less efficient without intermittent sonication. For instance, we obtain only 5-fold PrP^Sc^ amplification in each 3-day round of shaking propagation compared to >10-fold PrP^Sc^ amplification in each 1-day round of sPMCA propagation [33]. It is possible that this slower, less-efficient replication process may be more conducive for propagating metastable wild-type and mutant PrP^Sc^ conformations because there is less chance for selection and growth of a single thermodynamically favored conformer over the course of multiple propagation rounds. Continuous shaking may create less selective pressure on PrP^Sc^ replication than sPMCA, and thereby provide a better platform for studying conformational diversity in purified prions. From a technical perspective, it is important to note that specific physical parameters such as shaking speed and orbit diameter are required to produce this permissive environment, and some shaking protocols cannot maintain metastable wild-type PrP^Sc^ conformations. In contrast, mutant PrP^Sc^ conformations appear to be less sensitive to changes in shaking parameters.

In conclusion, we have used continuous shaking-induced formation of purified prions as a favorable platform to reveal fundamental differences between the mechanisms employed by wild-type and mutant prions to generate conformational diversity.

## Materials and methods

### Ethics statement

The Guide for the Care and Use of Laboratory Animals of the National Research Council was strictly followed for animal experiments. The mouse bioassay experiment in this study was conducted in accordance with protocol supa.su.1 as reviewed and approved by Dartmouth College’s Institutional Animal Care and Use Committee, operating under the regulations/guidelines of the NIH Office of Laboratory Animal Welfare (assurance number A3259-01).

### Expression and purification of recPrP constructs

The generation of WT Mo PrP (23–230) [9], BV PrP (23–231) with the methionine polymorphism at residue 109 (M109) [13], D177N mouse PrP (23–230) [24], and E199K mouse PrP (23–230) [24] expression plasmids have been described previously. All plasmids used in this study were created by incorporation of amplified PrP DNA sequences into the pET-22b (+) expression vector and transformed into Rosetta (DE3) *E*. *coli* cells (Novagen, Madison, WI).

Cultures were grown in Terrific Broth under selection with ampicillin and chloramphenicol, and protein expression was induced by addition of 1 mM IPTG. WT mouse PrP was purified by the method of Wang *et al*. [9,11]. All other constructs were purified by the method of Makarava *et al*. [13,24,34] Lyophilized protein was resuspended in water and adjusted to a concentration of 0.12 mg/mL, as measured by A_280_, one day before use.

### Purification of phospholipid cofactor

Phospholipid cofactor was purified from either normal mouse brains or rabbit brains. Both purifications were performed according to the method of Deleault *et al*.[9], with the rabbit brain purification containing the following modifications to facilitate higher throughput production. A 10% (w/v) brain homogenate was prepared by processing 100 g frozen rabbit brains (Pel-Freez, Rogers, AR) in 900 mL of pre-chilled Buffer A (20 mM MOPS pH 7.0, 150 mM NaCl) with a Hurricane Pro 3.5 Peak HP Blender (Cuisinart, Stamford, CT.) on ice crush for 30s. Debris was removed by centrifugation for 60 sec at 200 x *g*. The post-nuclear supernatant was centrifuged for 45 min at 10,000 x *g*, and the resulting pellet was re-homogenized in 900 mL of pre-chilled Buffer A containing 3% (w/v) n-octyl-β-D-glucopyranoside (nOG) (Anatrace, Maumee, OH) using a Potter homogenizer, and subsequently stirred at room temperature for 45 min to allow for solubilization. Next, the homogenate was centrifuged at 38,400 x *g* for 120 min. The resulting supernatant was adjusted to 2 mM CaCl_2_ and 25 U/mL TurboNuclease (Accelagen, San Diego, CA) and incubated in a 37°C water bath for 60 min with intermittent mixing. Thermolysin (Sigma, St. Louis, MO) was added at a final concentration of 25 μg /ml, and the sample was incubated at 70°C for 80 min with intermittent mixing. Next, the sample was adjusted to 5 mM EDTA, and cooled on ice before being centrifuged for 40 min at 38,400 x *g*. The supernatant was then placed in cellulose ester dialysis tubing with a 3,500 MWCO (Repligen, Waltham, MA) and dialyzed at 4°C against distilled water for 4 days at 8°C in a home-built continuous-flow apparatus designed to maintain high transmembrane pressure. Following dialysis, the sample was again frozen at -80°C and thawed to improve recovery by centrifugation. A centrifugation step of 90 min at 100,000 x *g* resulted in a pellet of two phases. The upper phase was collected and washed twice by resuspension in an equal volume of deionized water and centrifugation for 90 min at 100,000 x *g*, again collecting only the upper pellet phase after each spin. After a final spin for 120 min at 100,000 x *g*, the resulting pellet was resuspended in deionized water to 1/5 of the original homogenate volume.

### *In vitro* propagation of wild-type recPrP^Sc^ by continuous shaking and sPMCA

Cocktails for *in vitro* PrP^Sc^ conversion were prepared as described by Noble *et al*. [24], with minor modifications. Reactions containing 6 μg/mL BV recPrP in conversion buffer (20 mM Tris, 135 mM NaCl, 5 mM EDTA pH 7.5, 0.15% (v/v) Triton X-100, pH 7.4) were supplemented with mouse or rabbit brain-derived phospholipid cofactor prepared as described above. Reactions were initially seeded with 10% brain homogenates of animals infected with RML, Me7, 301C, Sc237, 139H, or Hyper, and serially propagate d at a 20% (v/v) seeding ratio for minimum 26 rounds in an Ohaus ITHSBLTS shaker (Parsippany, NJ) with 3 mm orbit at 2,000 rpm. Each reaction was shaken for 72 hr at a temperature of 37°C before propagation to the next round. For specific experiments, a custom shaker with 8 mm orbit was used at 1,200 rpm.

### *In vitro* propagation of D177N and E199K mutant recPrP^Sc^

Cocktails for *in vitro* PrP^Sc^ conversion were prepared as described by Noble *et al*. [24], with minor modifications. Reactions containing 6 μg/mL Mo recPrP 23–230 (either D177N or E199K) in conversion buffer (20 mM Tris, 135 mM NaCl, 5 mM EDTA pH 7.5, 0.15% (v/v) Triton X-100, pH 7.4) were initially seeded with either PMCA-generated D177N Mo PrP^Sc^ [24] or conversion buffer and subsequently serially propagated at a 20% (v/v) seeding ratio. A later set of reactions was seeded with the shaking generated E199K Mo PrP^Sc^ formed after initial seeding with D177N Mo PrP^Sc^. Shaking was performed in either an Eppendorf MixMate shaker operating at 2,000 rpm (maximal speed) or a home-built machine with an 8 mm orbit shaking at approximately 1,200 rpm. Each reaction was shaken for 72 hr at a temperature of 37°C before propagation to the next round.

### Proteinase K digestion of PrP^Sc^

PrP^Sc^ samples produced *in vitro* were treated with 20 μg/mL PK at 37°C for 30 min. Brain homogenates (10% (w/v) in PBS) from experimentally infected brains were digested in a reaction containing 50 μg/mL proteinase K (PK), 0.5% (v/v) Tween-20, 0.5% (v/v) NP-40, and 0.5% (w/v) nOG at 37°C with shaking at 750 rpm for 1 hr. All digestion reactions were quenched by addition of 4 mM PMSF.

### Enzymatic Deglycosylation

75 μl brain homogenate (10% (w/v) in PBS) was PK digested as described above and diluted to 1 mL in PBS, 0.5% (vol/vol) Triton X-100 and centrifuged at 100,000 x *g* for 60 min at 4°C. The resulting pellet was resuspended in 20 μl 5x glycoprotein denaturation buffer (NEB, Ipswich, MA) and subject to three 30-sec bursts of sonication and boiled at 95°C for 10 min. Samples were then diluted in 80 μL water with repeated sonication and boiling. Samples were cooled to room temperature and 13 μL each of 10x G7 reaction buffer and 10% (vol/vol) NP-40 and 5 μL PNGase F (NEB, Ipswich, MA) was added to each sample. Samples were then incubated overnight at 37°C and digestion terminated with the addition of SDS sample buffer and boiling at 95°C for 10 min before being run on SDS-PAGE.

### Western blotting

To detect PrP^Sc^ and analyze electrophoretic mobility after digestion, samples were first boiled for 15 min in Laemmli SDS sample buffer (Bioland Scientific, Paramount, CA). Samples were PK-digested unless otherwise indicated (-PK). SDS-PAGE and Western blotting were performed as described previously [35] using mAb 27/33 (epitope = 142–149 mouse numbering) primary and horseradish peroxidase-linked sheep anti-mouse secondary antibodies.

### Scrapie inoculation and neuropathology

Intracerebral inoculation and diagnosis of prion disease were performed as described [35] with the following modifications: Brain homogenate samples (10% (w/v) in PBS) were spun for 30 sec at 200 *x g* to remove nuclear debris, and the supernatant was collected and diluted in PBS + 1% BSA to be used as inoculum. The inoculum volume used was 30 μL. RML and Me7 were gifts from the Prusiner Lab (UCSF) and 301C was a gift from the Soto Lab (UTHealth McGovern School of Medicine). All strains were passaged in CD-1 mice prior to use. Female C57BL mice were obtained from Charles River Laboratories (Wilmington, MA, USA) and inoculated between 4–5 weeks of age.

Brains were removed rapidly at the time of sacrifice using new, sterile-packaged dissection instruments and disposable surfaces to avoid cross-contamination. They were immersion-fixed in 10% buffered formalin for 2–30 days, cut into ~3 mm thick sagittal sections, and placed in a tissue-processing cassette. Cassettes were treated with 88% formic acid for 1 hr, and then stored in PBS. The tissue was processed for paraffin embedding, and representative slides were stained with hematoxylin and eosin (H&E). Immunohistochemistry was performed on de-paraffinized slides using 2 μg/ml 27/33 anti-PrP mAb for 30 min at room temperature following citrate antigen retrieval and a Biocare (Concord, CA) Mouse on Mouse development kit.

## Supporting information

S1 Fig Comparison of input brain homogenate seeds and recombinant shaking products derived from various mouse and hamster strains. Western blot comparing migration of PrP^Sc^ molecules in prion-infected brain homogenates (Seed) and either bank vole (BV) or mouse (Mo) recombinant products of Ohaus serial shaking reactions shown in Figs 1 and 3. All samples were subjected to PK digestion. The equivalent of 10 μL 10% w/v brain homogenate and 25 μL of shaking reaction product were loaded for visualization.
(TIF)

S2 Fig Bioassay of serial shaking-propagated Mo recPrPSc molecules. Kaplan*-*Meier survival plots for mice inoculated intracerebrally with various inocula, as indicated. Me7-seeded shaking propagated = blue circles, 301C-seeded shaking propagated = red triangles.
(TIF)

S3 Fig PrPSc molecules in brains of prion-infected mice. Western blots of PK-digested brain homogenate samples prepared from mice inoculated with: brain-derived prions (Input), samples serially propagated for 26 rounds by continuous shaking in a substrate cocktail containing BV recPrP plus purified phospholipid cofactor (Shaking), samples serially propagated for 18 rounds by sPMCA in a substrate cocktail containing BV recPrP plus purified phospholipid cofactor (sPMCA), or samples serially propagated for 26 rounds by continuous shaking in a substrate cocktail containing BV recPrP without cofactor (Mock). Serially propagated samples were initially seeded either with Me7 or 301C as indicated. All samples in both blots were digested with PK. All samples in the lower blot were also treated with PNGase F to remove N-linked glycans.
(TIF)

S4 Fig Selective patterns of vacuolation in infected mice. Images of specific brain regions in hematoxylin- & eosin-stained brain sections taken from mice inoculated with either 301-seeded or Me7-seeded, shaking-propagated Mo recPrP^Sc^ molecules, as indicated.
(TIF)

S5 Fig Effect of increasing shaking speed on converged BV recPrPSc variants. Western blots showing subsequent serial propagation of the adapted BV recPrP^Sc^ variants shown in Fig 4 in shaking reactions in a commercial Ohaus shaker with 3 mm orbit at 2,000 rpm. All reactions were propagated in a substrate cocktail containing BV recPrP plus purified phospholipid cofactor. Reactions were initially seeded with RML, Me7, 301C, or Hyper as indicated. Following 5 rounds of serial propagation in a custom-made shaker with 8 mm orbit at 1,200 rpm, adapted conformers were subsequently propagated for 5 rounds in an Ohaus shaker with 3 mm orbit at 2,000 rpm. Reference protein-only PrP^Sc^ (PO) and cofactor PrP^Sc^ (Cof) samples are included on each blot for MW comparison. (-PK) = samples not subject to proteinase K digestion; all other samples were proteolyzed.
(TIF)
