# Supplementary figures and images for: Conformational diversity in purified prions produced in vitro
Source: PLoS Pathog. 2023 Jan 10;19(1):e1011083. doi: 10.1371/journal.ppat.1011083 (PMC9870145; doi:10.1371/journal.ppat.1011083)

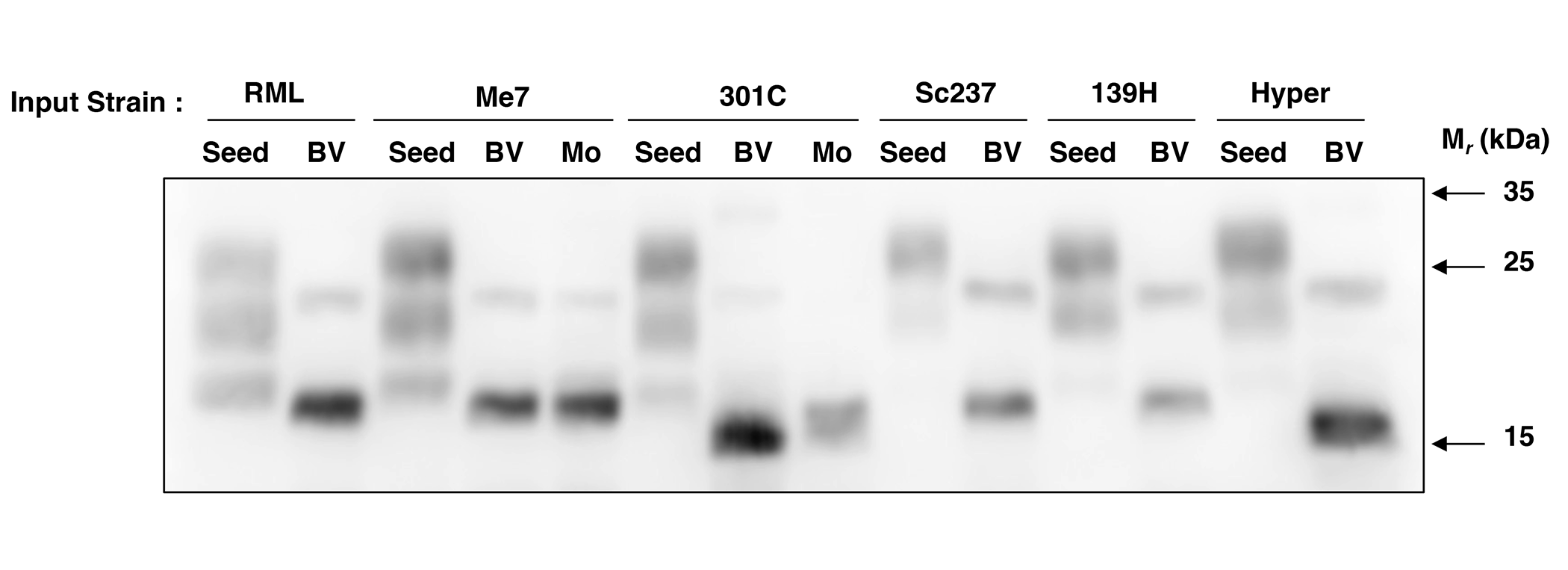

Supplement: S1 Fig — Western blot comparing migration of PrPSc molecules in prion-infected brain homogenates (Seed) and either bank vole (BV) or mouse (Mo) recombinant products of Ohaus serial shaking reactions shown in Figs 1 and 3. All samples were subjected to PK digestion. The equivalent of 10 μL 10% w/v brain homogenate and 25 μL of shaking reaction product were loaded for visualization. (TIF) [file ppat.1011083.s001.tif]

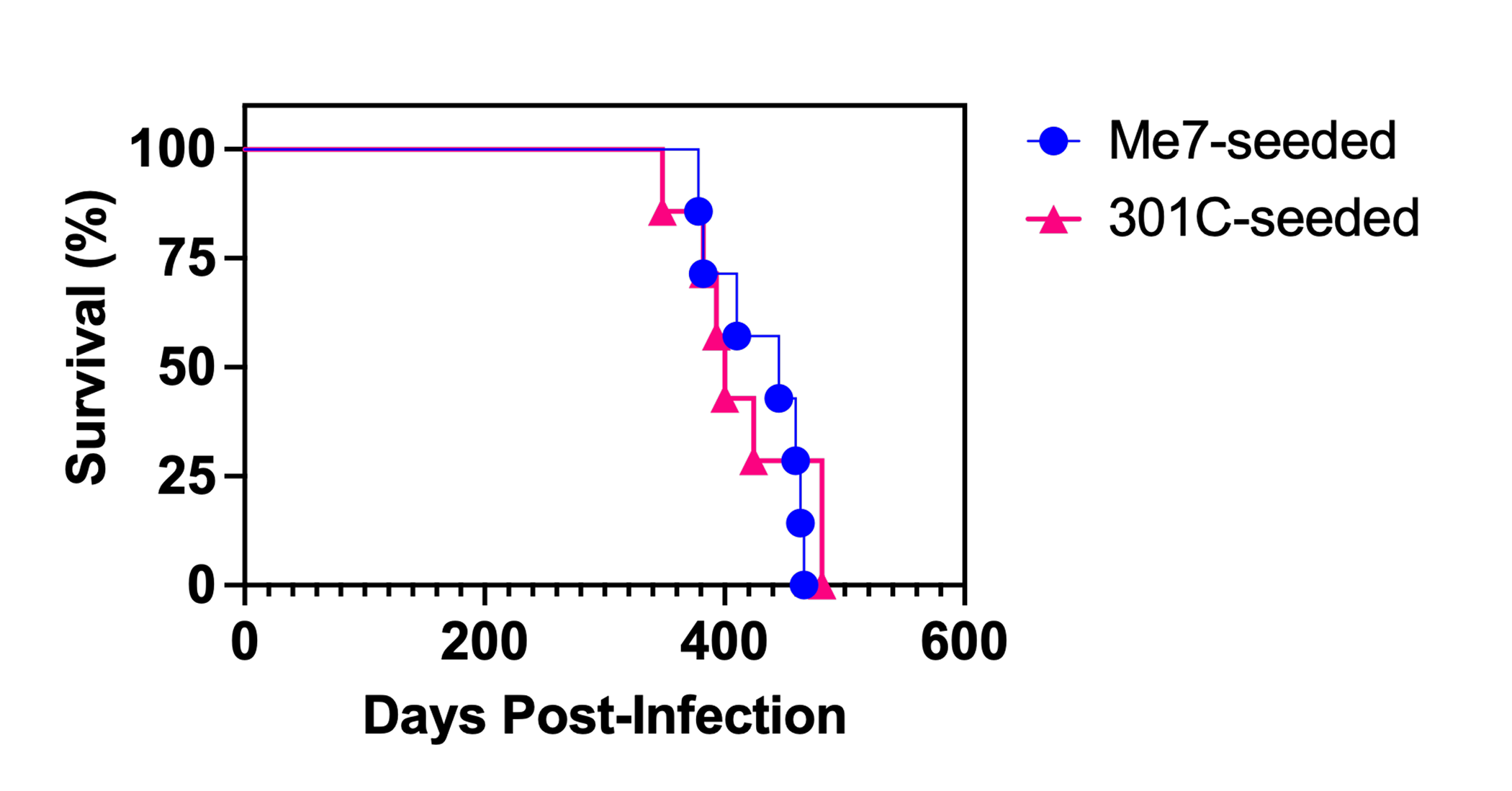

Supplement: S2 Fig — Kaplan-Meier survival plots for mice inoculated intracerebrally with various inocula, as indicated. Me7-seeded shaking propagated = blue circles, 301C-seeded shaking propagated = red triangles. (TIF) [file ppat.1011083.s002.tif]

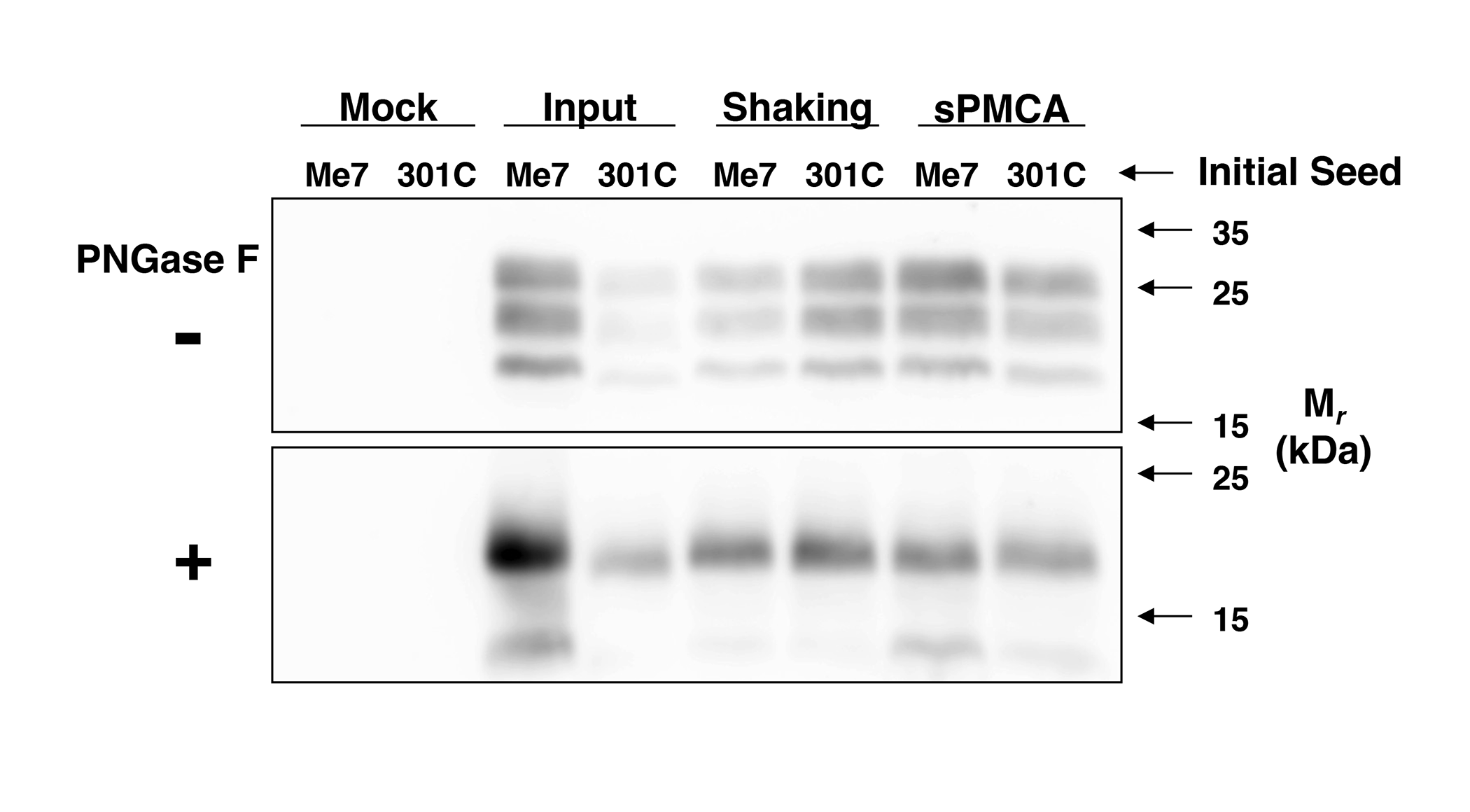

Supplement: S3 Fig — Western blots of PK-digested brain homogenate samples prepared from mice inoculated with: brain-derived prions (Input), samples serially propagated for 26 rounds by continuous shaking in a substrate cocktail containing BV recPrP plus purified phospholipid cofactor (Shaking), samples serially propagated for 18 rounds by sPMCA in a substrate cocktail containing BV recPrP plus purified phospholipid cofactor (sPMCA), or samples serially propagated for 26 rounds by continuous shaking in a substrate cocktail containing BV recPrP without cofactor (Mock). Serially propagated samples were initially seeded either with Me7 or 301C as indicated. All samples in both blots were digested with PK. All samples in the lower blot were also treated with PNGase F to remove N-linked glycans. (TIF) [file ppat.1011083.s003.tif]

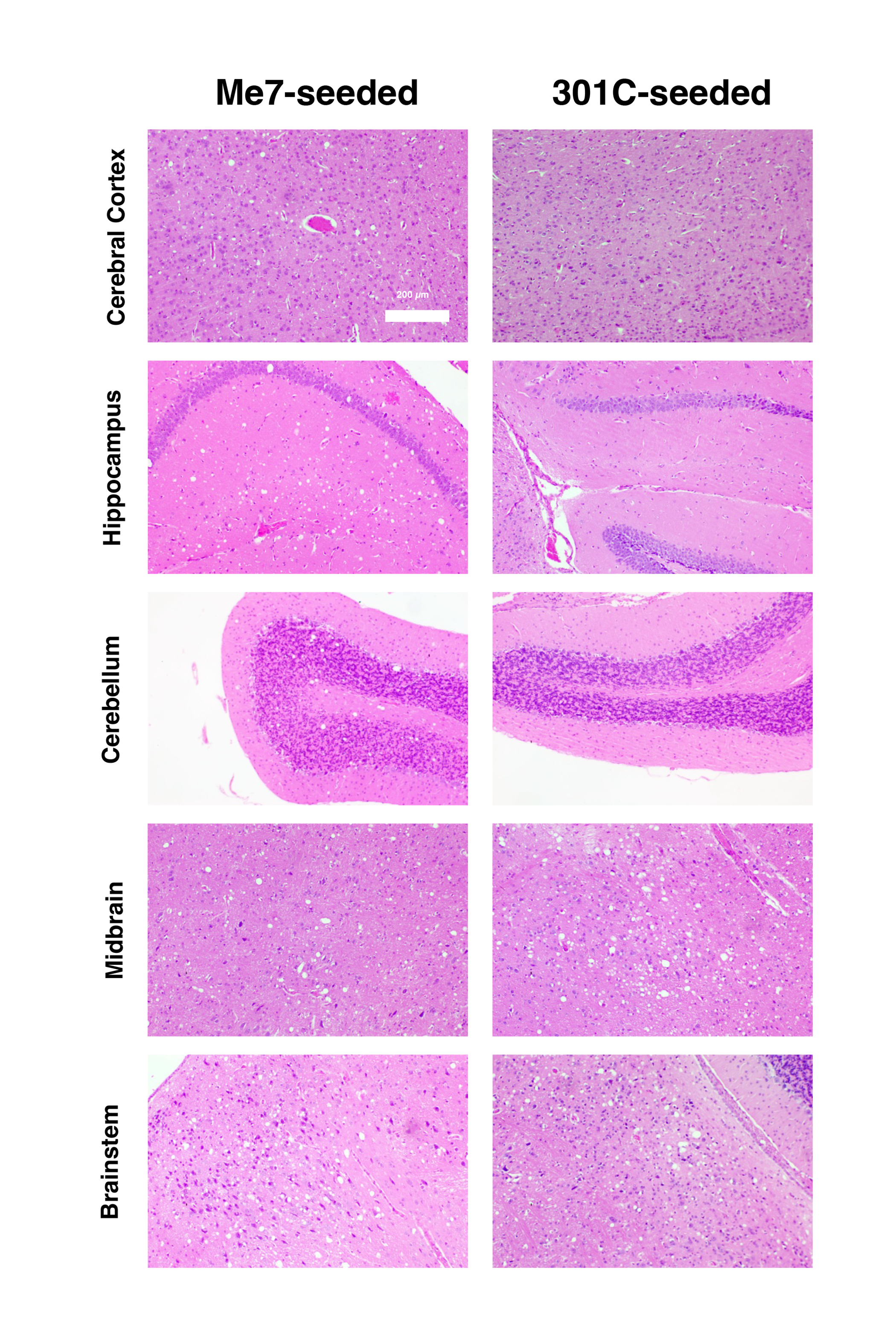

Supplement: S4 Fig — Images of specific brain regions in hematoxylin- & eosin-stained brain sections taken from mice inoculated with either 301-seeded or Me7-seeded, shaking-propagated Mo recPrPSc molecules, as indicated. (TIF) [file ppat.1011083.s004.tif]

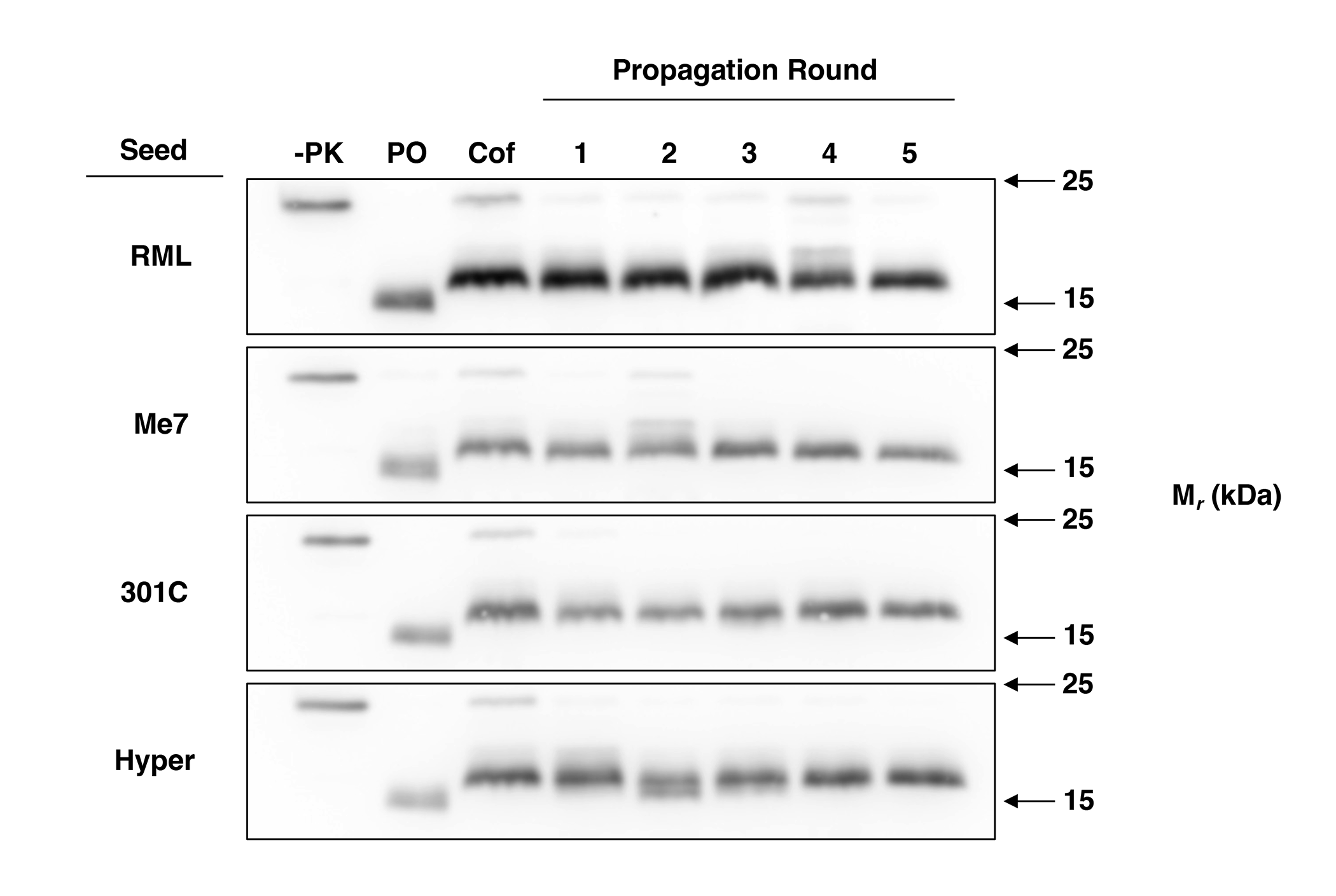

Supplement: S5 Fig — Western blots showing subsequent serial propagation of the adapted BV recPrPSc variants shown in Fig 4 in shaking reactions in a commercial Ohaus shaker with 3 mm orbit at 2,000 rpm. All reactions were propagated in a substrate cocktail containing BV recPrP plus purified phospholipid cofactor. Reactions were initially seeded with RML, Me7, 301C, or Hyper as indicated. Following 5 rounds of serial propagation in a custom-made shaker with 8 mm orbit at 1,200 rpm, adapted conformers were subsequently propagated for 5 rounds in an Ohaus shaker with 3 mm orbit at 2,000 rpm. Reference protein-only PrPSc (PO) and cofactor PrPSc (Cof) samples are included on each blot for MW comparison. (-PK) = samples not subject to proteinase K digestion; all other samples were proteolyzed. (TIF) [file ppat.1011083.s005.tif]
